# Inflammation-mediated SOD-2 upregulation contributes to epithelial-mesenchymal transition and migration of tumor cells in aflatoxin G_1_-induced lung adenocarcinoma

**DOI:** 10.1038/s41598-017-08537-2

**Published:** 2017-08-11

**Authors:** Li Yi, Haitao Shen, Mei Zhao, Peilu Shao, Chunping Liu, Jinfeng Cui, Juan Wang, Can Wang, Ningfei Guo, Lifei Kang, Ping Lv, Lingxiao Xing, Xianghong Zhang

**Affiliations:** 1 Department of Pathology, The Second Hospital, Hebei Medical University, Shijiazhuang, China; 2 Lab of Pathology, Hebei Medical University, Shijiazhuang, China; 3 Department of Dermatology,The Third Hospital, Hebei Medical University, Shijiazhuang, China; 4 Department of Pharmacology, Hebei Medical University, Shijiazhuang, China

## Abstract

Tumor-associated inflammation plays a critical role in facilitating tumor growth, invasion and metastasis. Our previous study showed Aflatoxin G_1_ (AFG_1_) could induce lung adenocarcinoma in mice. Chronic lung inflammation associated with superoxide dismutase (SOD)-2 upregulation was found in the lung carcinogenesis. However, it is unclear whether tumor-associated inflammation mediates SOD-2 to contribute to cell invasion in AFG1-induced lung adenocarcinoma. Here, we found increased SOD-2 expression associated with vimentin, α-SMA, Twist1, and MMP upregulation in AFG_1_-induced lung adenocarcinoma. Tumor-associated inflammatory microenvironment was also elicited, which may be related to SOD-2 upregulation and EMT in cancer cells. To mimic an AFG1-induced tumor-associated inflammatory microenvironment *in vitro*, we treated A549 cells and human macrophage THP-1 (MΦ-THP-1) cells with AFG_1_, TNF-α and/or IL-6 respectively. We found AFG_1_ did not promote SOD-2 expression and EMT in cancer cells, but enhanced TNF-α and SOD-2 expression in MΦ-THP-1 cells. Furthermore, TNF-α could upregulate SOD-2 expression in A549 cells through NF-κB pathway. Blocking of SOD-2 by siRNA partly inhibited TNF-α-mediated E-cadherin and vimentin alteration, and reversed EMT and cell migration in A549 cells. Thus, we suggest that tumor-associated inflammation mediates SOD-2 upregulation through NF-κB pathway, which may contribute to EMT and cell migration in AFG_1_-induced lung adenocarcinoma.Introduction.

## Introduction

Aflatoxins (AF), including AFB_1_, AFG_1_, and AFM_1_ are a type of mycotoxins, which are hepatotoxic and strongly carcinogenic, produced by certain molds of dietary staples in Asia and Africa^1, 2^. Our previous epidemiological study showed that aflatoxin G_1_ (AFG_1_) was one of the most frequently detected mycotoxins in contaminated grains and foodstuffs in areas of northern China with high incidence of lung and esophageal cancer^3^. Recently, we found that oral administration of AFG_1_ for 6 months induced chronic lung inflammation in BALB/c mice^4^. We prolonged the survival of the mice in the above model for another 6 months, and found that AFG_1_ induced lung adenocarcinoma in BALB/c mice. The result indicates that AFG_1_-induced chronic inflammation at the pre-tumor stage may be associated with lung tumorigenesis.

Chronic inflammation upregulates ROS, which causes direct genotoxic effects, such as DNA damage, and contributes to tumor initiation, invasion, and migration^5^. Before the initiation of AFG_1_-induced lung adenocarcinoma, we also reported that AFG_1_-induced chronic inflammation may cause alveolar epithelial oxidative stress evidenced by the increase of manganese superoxide dismutase (SOD-2) expression^4^. SOD-2 is one of the primary cellular antioxidant enzymes, which is vital in the regulation of oxidative stress by catalyzing the conversion of superoxide to hydrogen peroxide^6^. Up-regulation of SOD2 was shown to contribute to the migration and invasion of tongue squamous cell carcinoma, salivary adenoid cystic carcinoma as well as ovarian clear cell carcinoma^7–9^. Increased expression of SOD-2 in lung cancer was associated with increased metastases^10^. However, it has been shown that the overexpression of SOD-2 inhibited the migration, invasion and growth of prostate cancer cells^11, 12^. Finley *et al*., reported SOD-2 upregulation was proposed as a tumor suppressor gene in breast cancers^13^. Thus, the role of SOD-2 in tumorigenesis has been widely investigated and is still being explored. Whether SOD-2 contributes to the migration and invasion of AFG_1_-induced lung adenocarcinoma is unknown.

Several studies have shown that TNF-α/NF-κB pathway in tumor-associated inflammatory microenvironment is important to trigger epithelial-mesenchyme transition (EMT) to facilitate cancer cell motility, invasiveness, and metastatic potential^14, 15^. We reported that NF-κB and *p*-STAT3 expression as well as production of TNF-α and interleukin (IL)-6 were increased in AFG_1_-induced inflamed lung tissues^4^. However, it remains to be elucidated whether the tumor-associated inflammatory microenvironment is induced to promote EMT and migration in AFG_1_-induced lung adenocarcinoma. Kinugasa *et al*. reported that SOD2 is upregulated through NF-κB pathway in well-defined transformed oral and esophageal human epithelial cell lines undergoing EMT^16^. If AFG_1_ induces tumor-associated inflammatory responses, there is a critical need to identify whether the tumor-associated inflammation mediates SOD-2 upregulation to contribute to EMT and migration in AFG_1_-induced lung adenocarcinoma.

The aim of the present study was to investigate whether AFG_1_-induced tumor-associated inflammation mediates SOD-2 upregulation to contribute to EMT and cancer cell invasion in AFG_1_-induced lung adenocarcinoma. First, we collected lung tissue samples from mice with AFG_1_-induced lung adenocarcinoma in our previous study. The expression of TNF-α, IL-6, and macrophage marker CD68, as well as E-cadherin, vimentin, Twist, matrix metalloproteinase (MMP)-2, MMP-9, and SOD-2 were examined in AFG_1_-induced lung adenocarcinoma. Then, A549 cells or MΦ-THP-1 cells were treated with AFG_1_, IL-6, and TNF-α to mimic an AFG_1_-induced inflammatory response *in vitro*. We examined the mechanism of SOD-2 upregulation as well as its role in EMT and migration of cancer cells. This study may provide new insights into the underlying mechanism of metastatic potential in AFG_1_-induced lung adenocarcinoma.

## Results

### Enhanced SOD-2 expression in AFG_1_-induced lung adenocarcinoma

**Figure 1:**
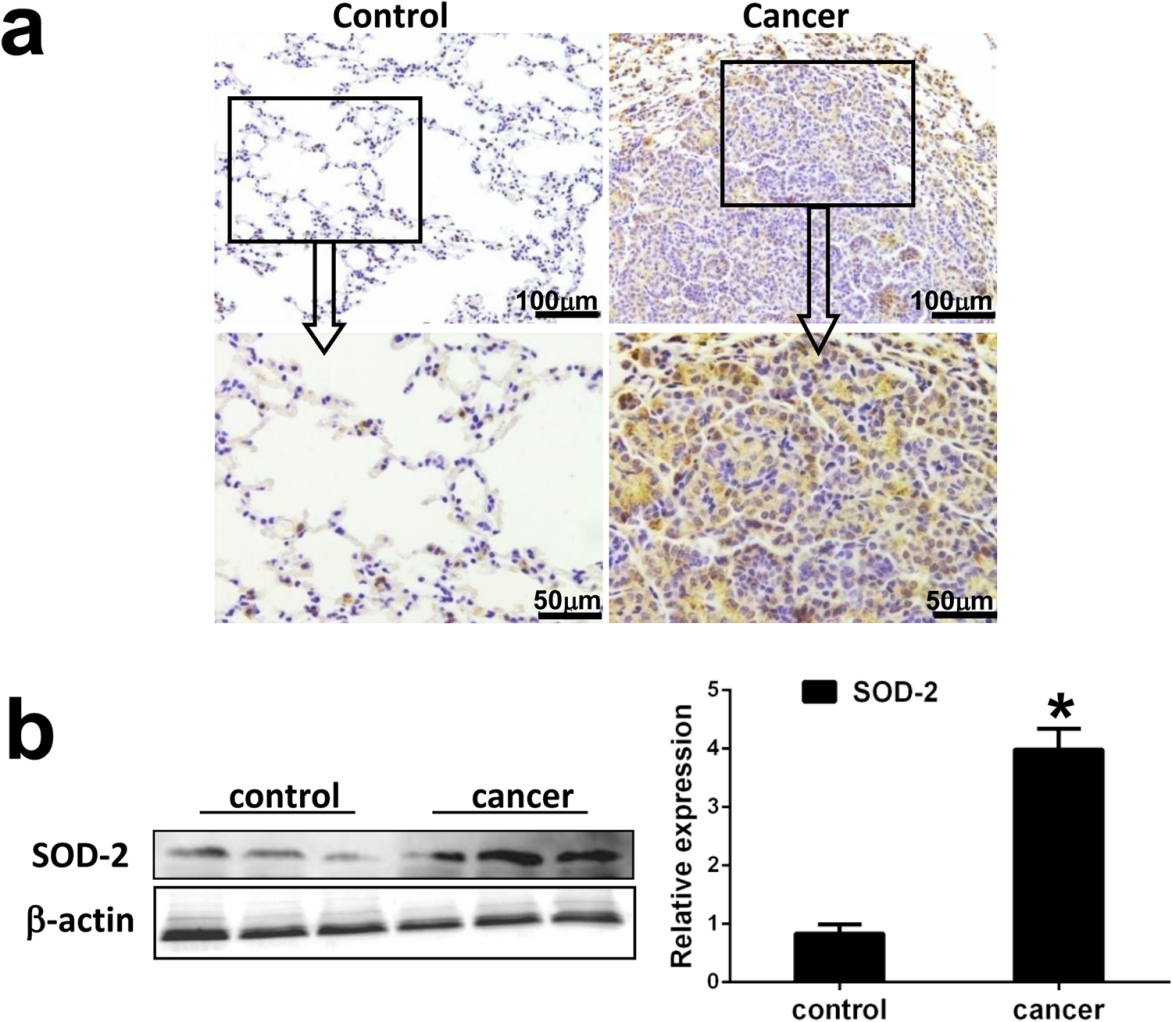
Enhanced SOD-2 expression is induced in AFG_1_-induced lung adenocarcinoma. (**a**) Paraffin sections of control lung tissues and AFG_1_-induced lung adenocarcinoma tissues from our previous study were prepared for immunohistochemical evaluation. Representative immunohistochemical staining of SOD-2 in AFG_1_-induced lung adenocarcinoma. (**b**) The total protein samples isolated from the lung adenocarcinoma or control lung tissues were collected from our previous study. The expression of SOD-2 at protein level from three separate control mice and AFG_1_-induced lung adenocarcinoma mice was shown by Western blot. Band intensity is coming from densitometry, and data was shown as mean ± SD (n = 3, *p < 0.05, compared to control group).

Immunohistochemical results showed diffused strong positive SOD-2 expressions in cytoplasm could be seen in AFG_1_-induced lung adenocarcinoma cells, while negative or weak staining of SOD-2 was observed in normal lung tissues. SOD-2 expression was significantly upregulated in AFG_1_-induced lung adenocarcinoma compared to that in control lung tissues (Fig. 1a). Western blot results further indicated that SOD-2 was upregulated in the lung tissues of AFG_1_-induced lung adenocarcinoma mice (Fig. 1b).

### EMT is induced in AFG_1_-induced lung adenocarcinoma

**Figure 2:**
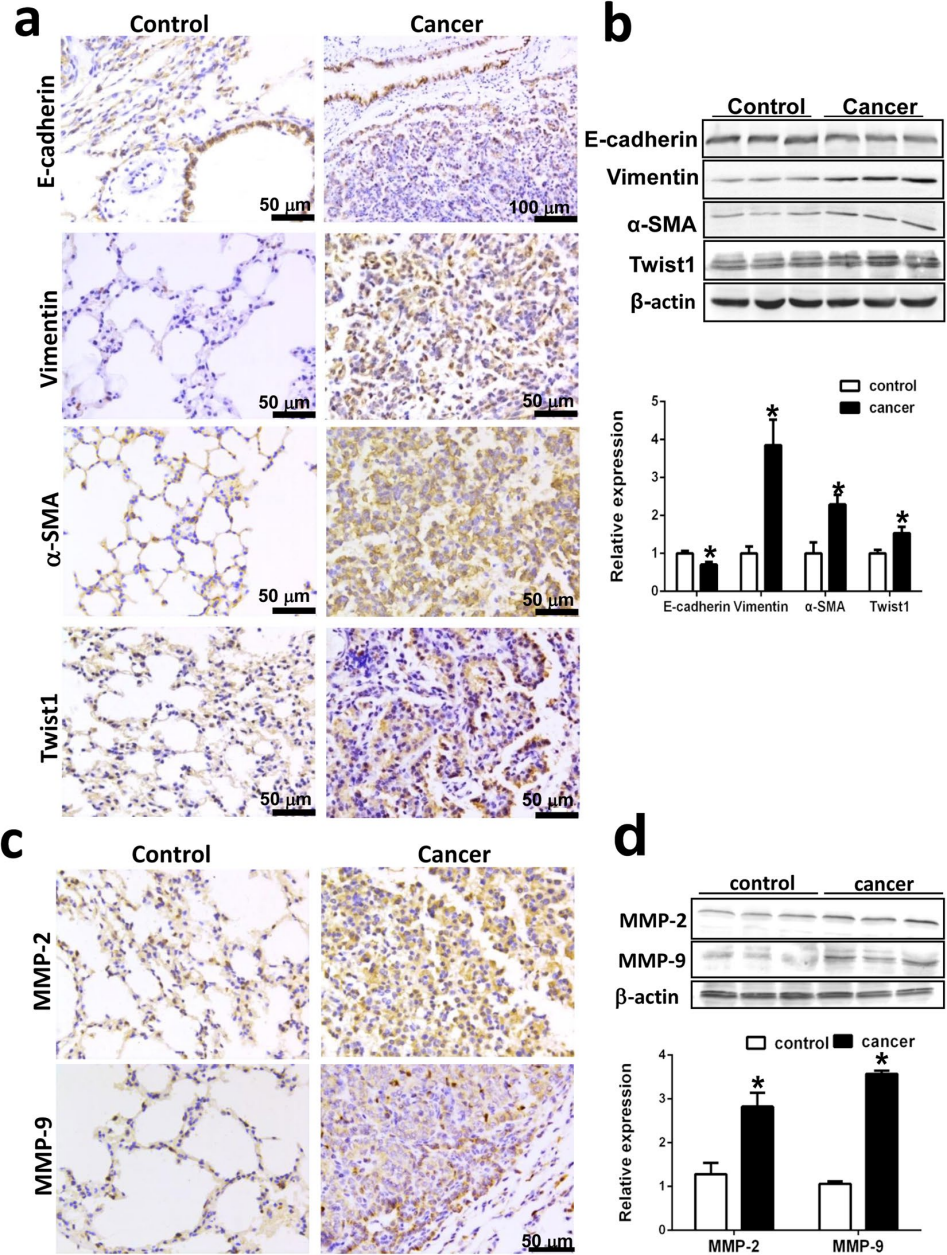
Epithelial-mesenchyme transition (EMT) is induced in AFG_1_-induced lung adenocarcinoma. Paraffin sections and the total protein samples isolated from the lung-adenocarcinoma or control lung tissues were collected from our previous study. (**a**) Representative immunohistochemical staining of E-cadherin, vimentin, α-SMA and twist1 in AFG_1_-induced lung adenocarcinoma. (**b**) The expression of E-cadherin, vimentin, α-SMA and twist1 at protein level was shown by Western blot. Band intensity is coming from densitometry, and data was shown as mean ± SD (n = 3, *p < 0.05, compared to control group). (**c**) Representative immunohistochemical staining of MMP2 and MMP9 in AFG_1_-induced lung adenocarcinoma. (**d**) The expression of MMP2 and MMP9 at protein level was shown by Western blot. Band intensity is coming from densitometry, and data was shown as mean ± SD (n = 3, *p < 0.05, compared to control group).

To further investigate the relation between SOD-2 overexpression and EMT, we examined the well-defined markers of EMT, including E-cadherin, vimentin, α-SMA, and Twist1 in AFG_1_-induced lung tumor. AFG_1_-induced lung adenocarcinoma expressed high levels of vimentin, α-SMA, and Twist1 in cancer cells (Fig. 2a). Positive expression of E-cadherin was observed in AFG_1_-induced lung adenocarcinoma, but E-cadherin expression was lower in lung adenocarcinoma compared to that in bronchial epithelial cells surrounding the adenocarcinoma or normal control (Fig. 2a). Western blot results showed elevated expression of vimentin, α-SMA, and Twist1, while lower expression of E-cadherin, in the lung tissues of mice with AFG_1_-induced lung adenocarcinoma (Fig. 2b). Those findings suggest that EMT of tumor cell is promoted in AFG_1_-induced lung adenocarcinoma.

Since EMT is also associated with the acquisition of migratory and invasive properties of tumor cells, we also examined the expression of MMP-2 and MMP-9. We found that AFG_1_-induced lung adenocarcinoma expressed high levels of MMP-2 and MMP-9 (Fig. 2c). Western blot results showed elevated expression of MMP-2 and MMP-9 in the lung tissues of mice with AFG_1_-induced lung adenocarcinoma (Fig. 2d). The results indicate that EMT of tumor cells is induced in AFG_1_-induced lung adenocarcinoma, which may increase the metastatic potential of tumor cells.

### Tumor-associated inflammatory microenvironment is elicited in AFG_1_- induced lung adenocarcinoma

**Figure 3:**
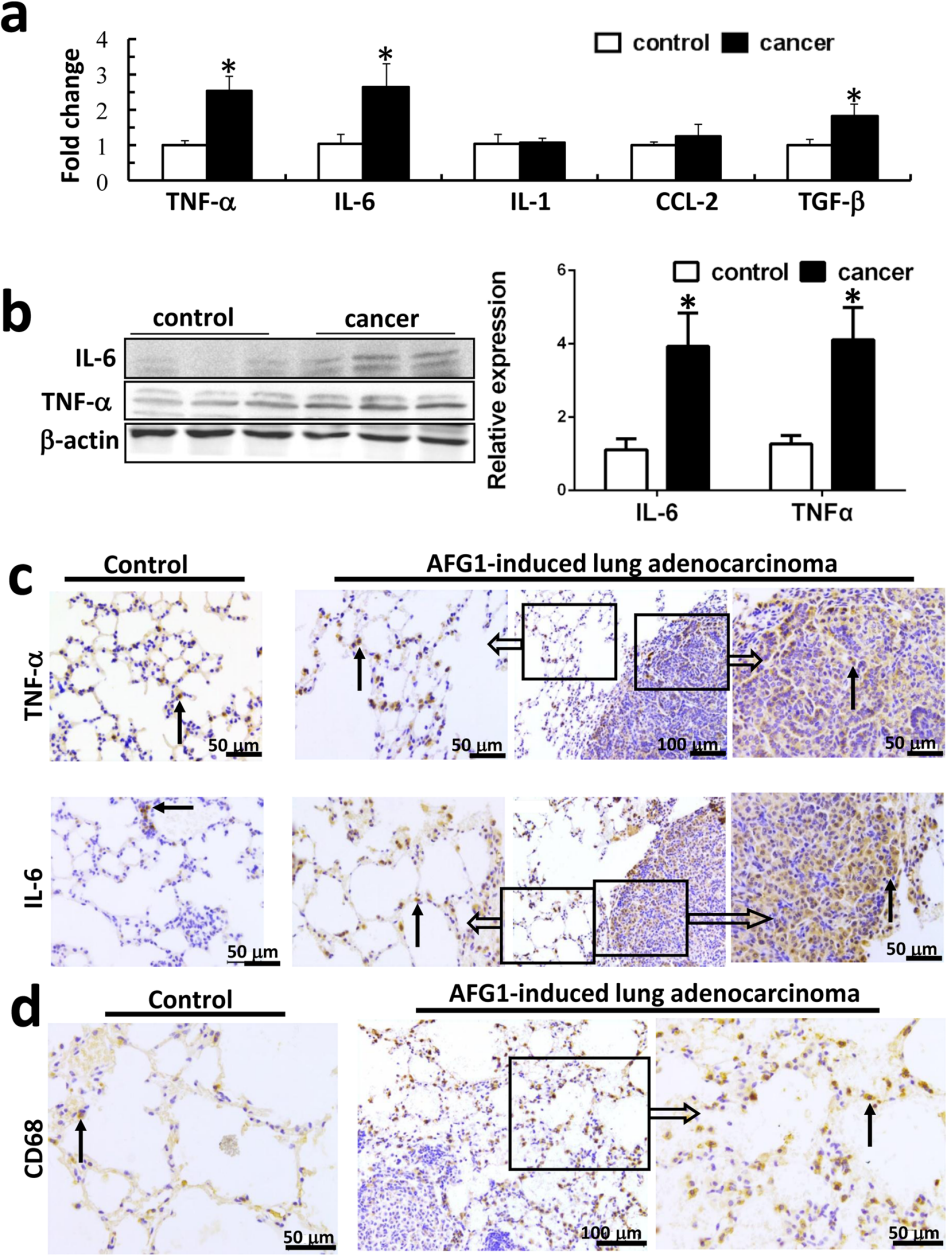
Tumor-associated inflammatory microenvironment is elicited in AFG_1_-induced lung adenocarcinoma. The RNA and protein samples as well as paraffin sections isolated from the lung-adenocarcinoma or control lung tissues were collected from our previous study. (**a**) Several cytokines and chemokines expression at mRNA level were determined by real-time PCR. Data was shown as mean ± SD (n = 4, *p < 0.05, compared to control group). (**b**) The expression of IL-6 and TNF-α at protein level was shown by Western blot. Band intensity is coming from densitometry, and data was shown as mean ± SD (n = 3, *p < 0.05, compared to control group). (**c**) Representative immunohistochemical staining of IL-6 and TNF-α in AFG_1_-induced lung adenocarcinoma. (**d**) Representative immunohistochemical staining of CD68 in lung tissues close to lung adenocarcinoma and control lung tissues. Black arrow points to positive cell.

qRT/PCR and Western blot results showed that the expression of TNF-α and IL-6 at the mRNA and protein levels were significantly upregulated in AFG_1_-induced lung adenocarcinoma tissues (Fig. 3a and b). To further explore which cells contribute to TNF-α and IL-6 production, we also detected TNF-α and IL-6 expression in AFG_1_-induced lung cancer cells as well as in tumor-adjacent tissues by immunohistochemical staining. Immunohistochemically, higher level of TNF-α and IL-6 expressions in cancer cell were observed in AFG_1_-induced lung adenocarcinoma (Fig. 3c). At the same time, there were more TNF-α and IL-6 positive cells in tumor-adjacent tissues than that in control lung tissues (Fig. 3c), which suggests that tumor-adjacent cells surrounding the adenocarcinoma may also contribute to TNF-α and IL-6 production. Furthermore, we revealed an increase in the numbers of infiltrating CD68^+^ macrophages in the alveolar septum surrounding the lung adenocarcinoma (Fig. 3d).

The results suggest that AFG_1_-induced tumor-associated inflammatory microenvironment consists of cytokines production by cancer cells and the surrounding tumor-adjacent cells as well as infiltrating macrophages.

### Upregulation of SOD-2 in cancer cells is dependent on the inflammation-activated NF-κB pathway

**Figure 4:**
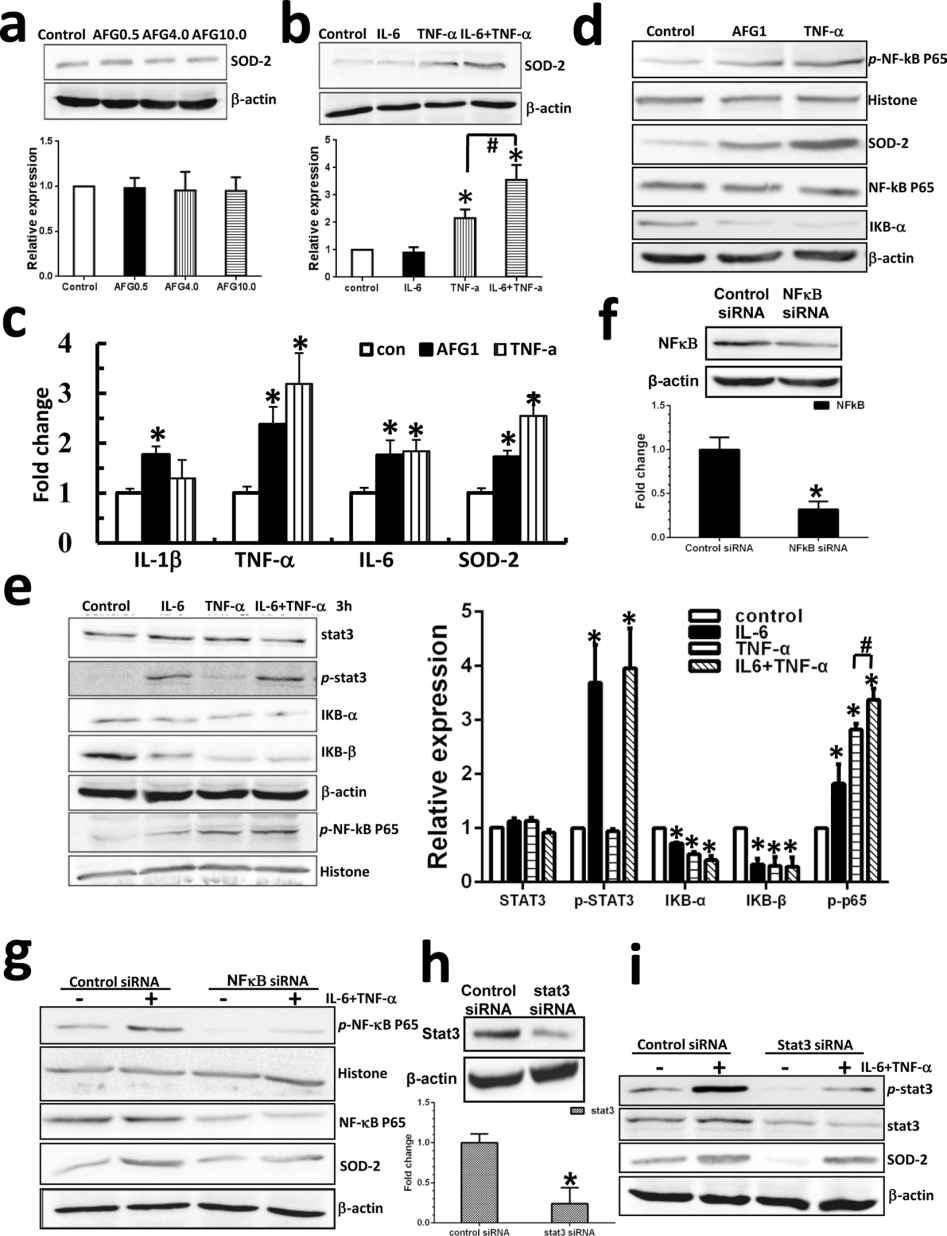
Up-regulation of SOD-2 in cancer cells is dependent on inflammation-activated NF-κB pathway. To mimic an AFG_1_-induced tumor-associated inflammatory response *in vitro*, we treated human lung adenocarcinoma cell line A549 and human macrophage cell line MΦ-THP-1 with AFG_1_ and/or TNF-α and IL-6. (**a**,**b**) After AFG_1_ or TNF-α and IL-6 treatment for 24 h, the expression of SOD-2 in A549 cell was detected by Western blot. Data was shown as mean ± SD (three experiments, *p < 0.05, compared to control group). (**c**) MΦ-THP-1 cells were treated with AFG_1_ or TNF-α for 24 h, and the expression of TNF-α, IL-6, IL-1 and SOD-2 at mRNA level were determined by real-time PCR. Data was shown as mean ± SD (three experiments, *p < 0.05, compared to control group). (**d**) After AFG_1_ or TNF-α treatment for 24 h, the expression of SOD-2 as well as the activation of NF-κB pathway in MΦ-THP-1 cells was measured by Western blot. (**e**) A549 cells were treated with TNF-α, IL-6, or TNF-α plus IL-6 for 3 hours, and the activation of NF-κB and STAT3 pathways was measured by Western blot. Data was shown as mean ± SD (three experiments, *p < 0.05, compared to control group, ^#^ *p* < 0.05, compared to TNF-α alone). The expression of NF-κB or STAT3 at mRNA and protein level was significant inhibited after transfection with NF-κB siRNA (**f**) or STAT3 siRNA (**h**) in A549 cells. After 24 h of TNF-α plus IL-6 treatment in A549 cells tansfected with NF-κB siRNA (**g**) or STAT3 siRNA (**i**), the expression of SOD-2 was detected by Western blot.

To establish the mechanism of SOD-2 upregulation in AFG_1_-induced lung tumor inflammatory microenvironment, A549 cells were treated with different concentrations of AFG_1_, IL-6, TNF-α, and TNF-α plus IL-6. We first explored whether AFG_1_ affects SOD-2 expression, and found that different concentrations of AFG_1_ did not induce SOD-2 expression in A549 cells (Fig. 4a). But when A549 cells were treated with TNF-α or IL-6, TNF-α could upregulate SOD-2, while IL-6 did not affect SOD-2 expression in A549 cells (Fig. 4b). The expression of SOD-2 in A549 cells treated with TNF-α plus IL-6 was significantly higher than that treated with TNF-α alone, which suggests that IL-6 may enhance TNF-α-upregulated SOD-2 expression (Fig. 4b). Then, we treated human macrophage cell line THP-1 (MΦ-THP-1) with AFG_1_ or TNF-α to further explore the mechanism of SOD-2 upregulation, and found AFG_1_ as well as TNF-α significantly upregulated IL-1, IL-6, TNF-α, and SOD-2 expression at mRNA level in MΦ-THP-1 cells (Fig. 4c). Western blot results showed that AFG_1_ could activate NF-κB signaling pathway and upregulate SOD-2 expression in MΦ-THP-1 cells (Fig. 4d). Though AFG_1_ could not directly promote SOD-2 expression in pulmonary adenocarcinoma cells, but stimulated macrophages to produce TNF-α and IL-6. Those cytokines may contribute to AFG_1_-induced inflammatory responses as well as SOD-2 upregulation in AFG_1_-induced lung adenocarcinoma.

Furthermore, we found treatment of TNF-α plus IL-6 for 3 hours significantly activated NF-κB and STAT3 signaling pathway in A549 cells (Fig. 4e). Combination of TNF-α plus IL-6 treatment induced higher level expression of *p*-NF-κB compared with TNF-α alone, suggesting that IL-6 could enhance TNF-α-mediated NF-κB activation (Fig. 4e). The NF-κB and STAT3 pathways were blocked by siRNA in A549 cells, confirmed by real-time PCR and western blot (Fig. 4f,h). We found NF-κB knockdown significantly inhibited SOD-2 upregulation in the cells treated with TNF-α plus IL-6, while blocking STAT3 did not affect SOD-2 upregulation (Fig. 4g,i). This suggests that the NF-κB pathway in AFG_1_-induced lung tumor inflammatory microenvironment may play a key role in upregulating SOD-2 expression.

### TNF-α working with IL-6, not AFG_1_, induced EMT and migration in lung cancer cells

**Figure 5:**
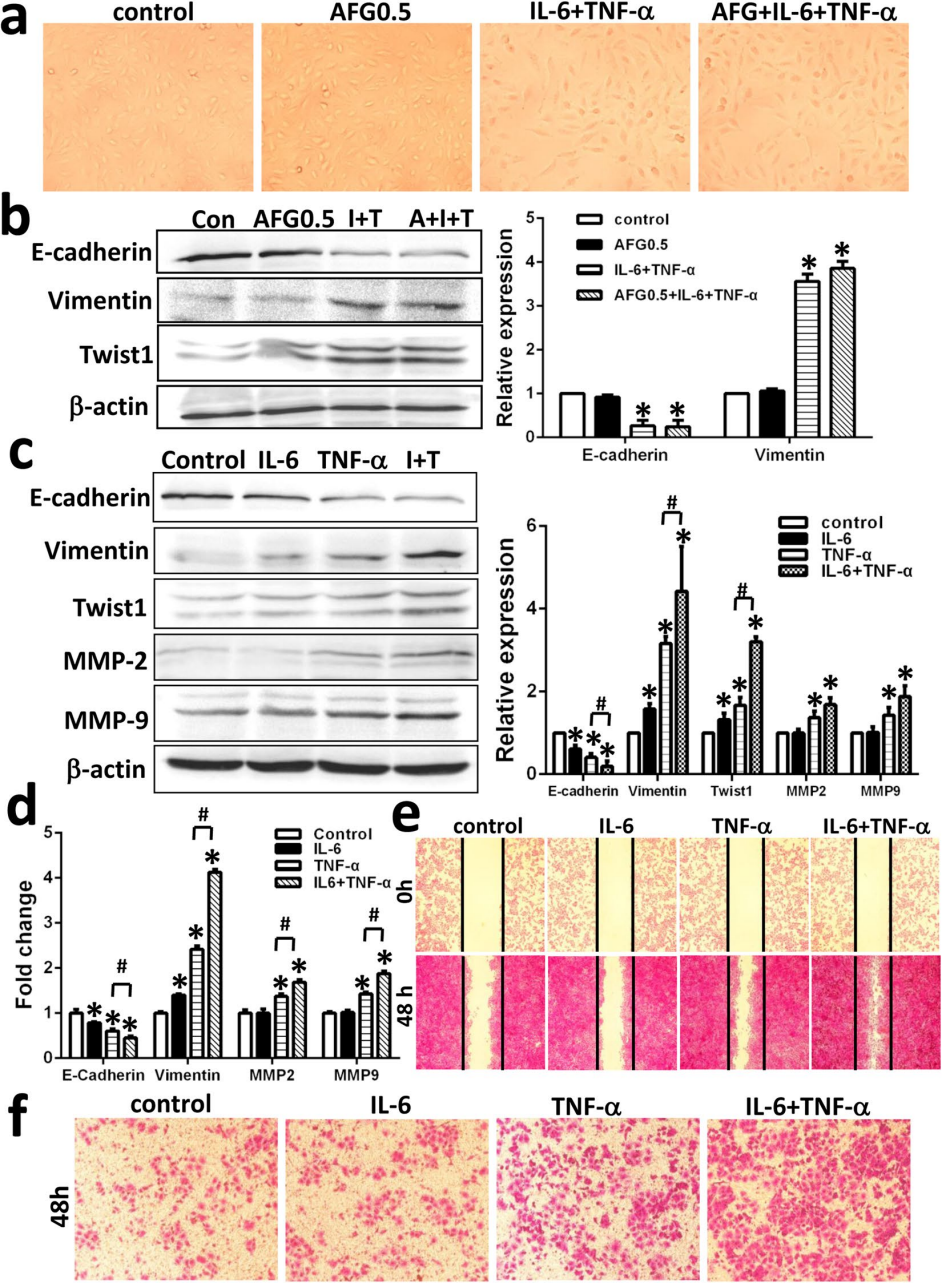
TNF-α working with IL-6, not AFG_1_, induced EMT and migration in lung cancer cells. A549 cell were treated with AFG_1_ and/or TNF-α plus IL-6 for 72 hours. (**a**) Morphological changes of A549 cell was observed under light microscopy. (**b**) The expression of E-cadherin, vimentin and twist1 in A549 cell was detected by Western blot. Band intensity is coming from densitometry, and data was shown as mean ± SD (three experiments, *p < 0.05, compared to control). A549 cells were treated with TNF-α, IL-6, or TNF-α plus IL-6 for 72 hours. The expression of E-cadherin, vimentin, twist1, MMP2 and MMP9 in A549 cell was detected by Western blot (**c**) and qPCR (**d**). Data was shown as mean ± SD (three experiments, *p < 0.05, compared to control group; ^#^ *p* < 0.05, compared to TNF-α alone). After 48 h treatment, the migration ability of A549 cell was measured by wound healing migration assay (**e**) and transwell migration assay (**f**).

We treated A549 with AFG_1_ and/or TNF-α with IL-6 to investigate to the key contributor of EMT and migration in AFG_1_-induced lung adenocarcinoma. The combined treatment of TNF-α and IL-6 could induce morphological changes in A549 cells, characterized by fibroblast-like cells, while AFG_1_ did not cause EMT in the tumor cells (Fig. 5a). Furthermore, the combination of TNF-α and IL-6 downregulated the expression of E-cadherin and upregulated the expression of vimentin and Twist1 in A549 cells, while AFG_1_ did not (Fig. 5b). TNF-α significantly promoted EMT and migration in A549 cells compared with IL-6 treatment, which only affected E-cadherin, vimentin and Twist1 expression (Fig. 5c). Western blot and qPCR results also showed that combination of TNF-α plus IL-6 treatment induced higher level expression of vimentin, Twist1, MMP-2 and MMP-9 as well as lower level expression of E-cadherin in A549 cells compared with TNF-α alone (Fig. 5c and d).

The wound healing migration assay and transwell migration assay demonstrated that TNF-α working together with IL-6 significantly enhanced A549 cell migration compared to TNF-α or IL-6 alone (Fig. 5e and f).

Taken together, the results suggest that TNF-α working together with IL-6 could promote EMT and migration in A549 cells, and TNF-α is the key contributor of EMT and migration in AFG_1_-induced lung adenocarcinoma.

### SOD-2 contributes to TNF-α-mediated EMT and migration in A549 cells

**Figure 6:**
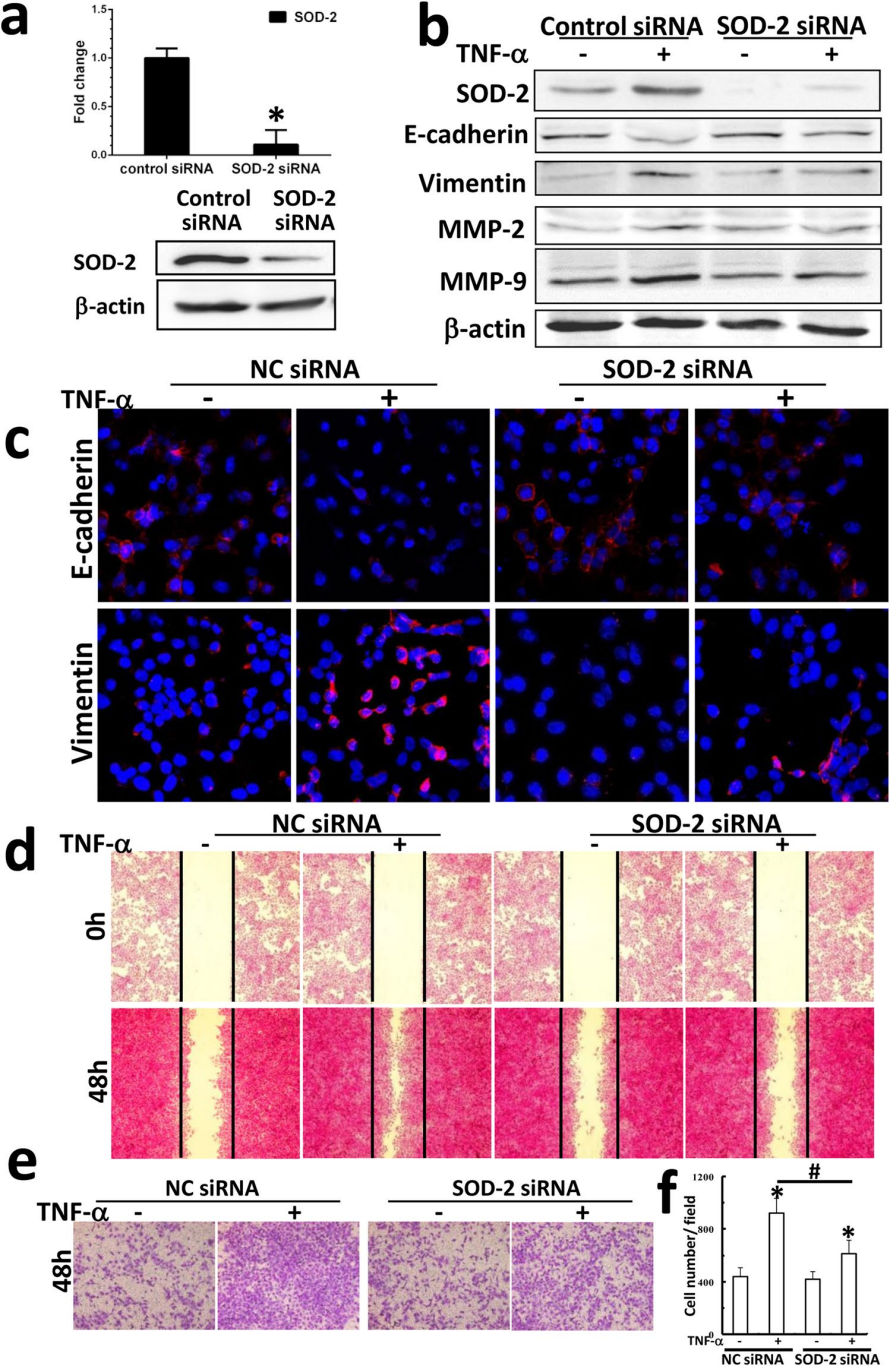
SOD-2 contributed to TNF-α-mediated EMT and migration in A549 cells. (**a**) The expression of SOD-2 at mRNA and protein level was significant inhibited after transfection with SOD-2 siRNA in A549 cells. (**b**) After 72 h of TNF-α treatment, the expression of SOD-2, E-cadherin, vimentin, MMP2 and MMP9 in A549 cell transfected with SOD-2 siRNA was detected by Western blot. (**c**) Representative immunofluorescent micrographs show the expression of E-cadherin and vimentin in SOD-2-knockdown cells. After TNF-α treatment for 48 hours, the migration ability of A549 cell transfected with SOD-2 siRNA was measured by wound healing migration assay (**d**) and transwell migration assay (**e**). (**f**) Cell number was determined by counting the positive cells number in higher field and data was shown as mean ± SD (three experiments, *p < 0.05, compared to control; ^#^ *p* < 0.05, control cells treated with TNF-α compared to SOD-2-knockdown cells treated with TNF-α).

To determine whether SOD-2 is involved in inflammation-mediated EMT and migration in cancer cells, SOD-2 was knockdown by siRNA in A549 cells, and then the cells were treated with TNF-α. We found that siRNA efficiently knocked down SOD-2 expression in A549 cells, confirmed by real-time PCR and Western blot (Fig. 6a). Blocking SOD-2 expression partly inhibited E-cadherin downregulation and vimentin upregulation induced by TNF-α (Fig. 6b). The EMT-like phenotypic changes were confirmed by detecting the expression of characteristic molecular markers using immunofluorescence. In SOD-2 knockdown cells, E-cadherin downregulation and vimentin upregulation in TNF-α-treated cells was partly inhibited (Fig. 6c). Thus, the suppression of SOD-2 partly reversed TNF-α-mediated EMT in A549 cells.

The blocking of SOD-2 expression also inhibited the upregulation of MMP-2 and MMP-9 in A549 cells treated with TNF-α, which suggest that SOD-2 upregulation may be responsible for cell migration (Fig. 6b). The wound healing assay showed that blocking SOD-2 expression partly inhibited cells migration in TNF-α-treated cells (Fig. 6d). Transwell assay results showed that cell numbers in bottom chamber of SOD-2 knockdown cells treated with TNF-α were lower than that of control cells (Fig. 6e and f). Those results support that blocking SOD-2 expression partly inhibited TNF-α-induced migration in A549 cells.

All above results suggest that SOD-2 upregulation upon inflammation responses may contribute to TNF-α-mediated EMT and migration in tumor cells in AFG_1_-induced lung adenocarcinoma.

## Discussion

Our recent studies showed that AFG_1_ could induce lung adenocarcinoma in mice models, which further supports studies showing that aflatoxins could induce lung cancer in experimental animals^4, 17, 18^. We also found that AFG_1_ induced chronic lung inflammatory responses associated with SOD-2 upregulation at the pre-tumor stage. In this study, we collected the lung tissues from AFG_1_-induced lung adenocarcinoma mice, and found increased SOD-2 expression in AFG_1_-induced lung adenocarcinoma. Our finding shows a similar conclusion as Chung-man’s, which reported that SOD-2 activity was increased in non-small cell lung cancer^19^. SOD-2 is overexpressed in alveolar type II epithelial cells and alveolar macrophages, which are essential in protecting lung tissues against free radicals^6^. Melloni reported that healthy smoking individuals with no lung dysfunction have less SOD in lung epithelial lining fluid, which suggesting that the increase in Mn SOD is associated with progression to lung dysfunction and/or cancer^20^. It has been showed that ingestion of foods high in antioxidant activity is associated with a decreased incidence of lung cancer^21^. Administration of food as antioxidants fails to prevent the development of lung cancer^22^. Thus, the role of SOD-2 upregulation in AFG_1_-induced lung adenocarcinoma is unclear.

Recently, several studies have reported that SOD-2 might favor tumor progression and migration^7–9, 23^. SOD2-dependent production of H_2_O_2_ leads to increased expression of MMP family members^7, 23, 24^. To further investigate the relation between SOD-2 overexpression and EMT, we examined the well-defined markers of EMT, including E-cadherin, vimentin, α-SMA, and Twist1 in AFG_1_-induced lung adenocarcinoma. We demonstrated a decrease of epithelial markers E-cadherin, and increase of mesenchymal cell markers vimentin and α-SMA, Twist1, known as an essential player in the aggressive phenotype of EMT, was also increased in AFG_1_-induced lung adenocarcinom^25^. The expression of MMP-2 and MMP-9, associated with tumor progression and metastasis, was also increased^23^. The results indicate that increased SOD-2 is associated with tumor progression and migration in AFG_1_-induced lung adenocarcinoma.

Kinugasa *et al*. used the transformed oral and esophageal human epithelial cell lines to show that inflammation may contribute to SOD2 upregulation^16^. Our previous studies have suggested that TNF-α and IL-6 are important mediators in AFG_1_-induced lung chronic inflammatory microenvironment^4^. However, whether a tumor-associated inflammatory microenvironment is induced in AFG_1_-induced lung adenocarcinoma is still unclear. In this study, we found increased TNF-α and IL-6 expression and macrophages infiltration in AFG_1_-induced lung adenocarcinoma. Furthermore, higher level of TNF-α and IL-6 expression was observed in cancer cell as well as the surrounding tumor-adjacent cells and immune cells, which suggests that AFG_1_-induced tumor-associated inflammatory microenvironment consists of cancer cells, tumor-adjacent cells, and infiltrating immune cells^14, 15, 26^. Those cells work together to produce pro-inflammatory and inflammatory cytokines, which may maintain an inflammatory environment to mediate SOD-2 upregulation in AFG_1_-induced lung adenocarcinoma.

To further investigate the mechanism of upregulation of SOD-2 in AFG_1_-induced lung tumor, we treated A549 cells and MΦ-THP-1 cells with AFG_1_, TNF-α and/or IL-6 to mimic the AFG_1_-induced tumor-associated inflammatory microenvironment *in vitro*. Furthermore, we found TNF-α upregulated SOD-2 expression in A549 cells; however, AFG_1_ and IL-6 did not induce SOD-2 upregulation. Combination of TNF-α plus IL-6 promoted SOD-2 expression compared with TNF-α alone, which suggests that IL-6 may enhanced TNF-α-upregulated SOD-2 expression in A549 cells. Then, we found both AFG_1_ and TNF-α could activate NF-κB pathway and promote TNF-α and IL-6 production as well as SOD-2 expression in MΦ-THP-1 cells. Though AFG_1_ could not directly promote SOD-2 expression in pulmonary adenocarcinoma cells, but did stimulate macrophages to produce TNF-α and IL-6. It has been shown that aflatoxin could increase the secretion of pro-inflammatory cytokines from the murine macrophage cell line, J774A.1^27^. Thus, those cytokines secreted by macrophages may contribute to AFG_1_-induced inflammatory responses as well as SOD-2 upregulation in AFG_1_-induced lung tumor. We also found that NF-κB pathway played a key role in upregulating SOD-2 expression in A549 cells. Several studies showed that activated STAT3 by IL-6 may account for constitutive activation of NF-κB pathway in some human cancer^28^. In this study, we found IL-6 treatment not only activated STAT3 pathway, but also showed some activating effect on NF-κB pathway. IL-6 also enhanced TNF-α-mediated NF-κB activation in A549 cells. Thus, it may be possible that IL-6-promoted SOD-2 expression in TNF-α-treated A549 cells may be due to enhanced activation of NF-κB pathway. Our previous study has shown that AFG_1_ could upregulate TNF-α expression in A549 cells, however AFG_1_ could not activated NF-κB pathway^4, 29^. Those findings suggest that the autocrine TNF-α produced by AFG_1_-activated A549 cells may be insufficient to drive NF-κB pathway activation to upregulate SOD-2 expression. Our findings strongly support a recent hypothesis where the inflammation amplifier was activated by the stimulation of cytokines, such as TNF-α and IL-6, resulting in the subsequent expression of tumor-related genes such as HIF1-α and SOD-2^30^. Taken together, we postulate that the inflammation-activated NF-κB pathway mainly contributes to the upregulation of SOD-2 in AFG_1_-induced lung tumor inflammatory microenvironment.

Chronic inflammation is a major activator of the metastatic cascade^31^. This tumor-associated inflammation plays a central role in the regulation of EMT, which contributes to cancer invasion and metastasis. However, Liu Y *et al*. have shown that the extract of cigarette smoke can induce EMT in human bronchial epithelial cells^32^. Before we further investigate the role of SOD-2 involved in EMT, we treated A549 cells with AFG_1_, TNF-α and IL-6 to explore the key contributor of EMT in AFG_1_-induced lung tumor. TNF-α showed higher ability to induce EMT than IL-6 in A549 cells, however AFG_1_ did not induce EMT. The findings indicate that TNF-α through NF-κB pathway may be the major contributor of EMT and migration in AFG_1_-induced lung tumor. To further explore whether inflammation-mediated SOD-2 is involved in EMT and migration in A549 cells, we examined EMT in SOD-2 siRNA-transfected cells that were treated with TNF-α. We found that the inhibition of SOD-2 with siRNA could reverse TNF-α-mediated EMT in A549 cells. Thus, the results further support that TNF-α-mediated SOD-2 upregulation through NF-κB pathway plays an important role to promote EMT in A549 cells. SOD2-dependent production of H_2_O_2_ leads to increased expression of MMP family members^7, 23, 24^. In agreement with previous studies, we found that the blocking of SOD-2 inhibited TNF-α-mediated upregulation of MMP-2 and MMP-9, as well as cell migration in A549 cells. The results indicate that SOD-2 may significantly favor inflammation-mediated EMT and migration of tumor cells in AFG_1_-induced lung adenocarcinoma. To the best of our knowledge, this is the first study to demonstrate that inflammation-mediated SOD-2 contributes to EMT in lung cancer cells.

Several studies have reported that aflatoxin could induce inflammatory reaction *in vivo*, and macrophages may play a key role in contributing to pro-inflammatory cytokines production^27, 33, 34^. Our recent studies also showed that AFG_1_ induced chronic lung inflammatory responses associated with increased macrophages infiltration and TNF-α expression as well as SOD-2 upregulation in lung tissues^4^. According to those findings, our results in this study could conclude that AFG_1_ may activate macrophages to produce cytokines, which contributes to inflammatory responses in AFG_1_-induced lung adenocarcinoma. TNF-α may be the major contributor of SOD-2 upregulation as well as EMT and migration in cancer cells. Thus, the inflammation-mediated SOD-2 through NF-κB pathway plays an important role in EMT and migration in tumor cells in AFG_1_-induced lung adenocarcinoma, which suggests a new mechanism of AFG_1_-induced lung carcinogenesis.

## Methods

### Materials

AFG_1_ and dimethyl sulfoxide (DMSO) were purchased from Sigma (St Louis, MO, USA). Rabbit anti-TNF-α was purchased from Bioworld (Cat# BS1857, Nanjing, China). Rabbit anti-IL-6 (Cat# DF6087), rabbit anti-MMP-2 (Cat# AF0577), rabbit anti-MMP-9 (Cat# AF5228), and rabbit anti-Twist1 (Cat# AF4009) were purchased from Affinity (OH, USA). Rat anti-CD68 (Cat# 137001) was purchased from BioLegend (San Digeo, CA, USA). Rabbit anti-α-SMA (Cat# ab5694) was purchased from abcam (Cambridge, MA, USA). Rabbit anti-IKB-α (Cat# 2246-s), rabbit anti-IKB-β (Cat# 3696-s), rabbit anti-nuclear factor (NF)-κB/p65 (Cat# 1546-1), rabbit anti-signal transducer and activator of transcription 3 STAT3 (Cat# 2236-1), and rabbit anti-SOD-2 (Cat# 2299-s) were purchased from Epitomics (CA, USA). Rabbit anti-E-cadherin (Cat# 3195P), vimentin (Cat#5741P), p-NF-κB/p-p65 (Cat# 3033P) and *p*-STAT3 (Cat# 9145S) were purchased from Cell signaling (Danvers, MA, USA). Cytokines IL-6 and TNF-α were purchased from Peprotech (Rocky Hill, NJ, USA).

### Immunohistochemical staining

Experimental protocols were approved by the Ethics Committee of Hebei Medical University of China. Paraffin sections of control lung tissue and AFG_1_-induced lung adenocarcinoma tissue from our previous study were prepared for immunological evaluation^4^. Briefly, the lung cancer induction study was as follows: BALB/c mice were oral administration of AFG_1_ (100 µg/kg), three times a week for 6 months. The control mice received 300 µl of saline and 0.6 µl of DMSO three times a week for 6 months. The experimental mice were sacrificed after another 6 months without AFG_1_ treatment, and lung adenocarcinoma incidence reached 26.7%.

The paraffin sections (5 µm) were immunohistochemically stained as described previously^4^. After blocking, the sections were incubated with TNF-α, IL-6, CD68, E-cadherin, vimentin, α-SMA, Twist1, SOD-2, MMP-2, and MMP-9 antibodies overnight at 4 °C (dilution 1:500). Visualization was achieved with peroxidase-labeled streptavidin-biotin and diaminobenzidine (DAB) staining. The positive cells were observed in high-power fields without blood vessels and bronchi by using a light microscope (400× objective).

### Cell culture and treatment

A549 cells were obtained from resource center of Peking Union Medical College Hospital of China and were approved by the Ethics Committee of Hebei Medical University of China. A549 cells were maintained in RPMI 1640 medium containing 10% fetal bovine serum and penicillin/streptomycin at 37 °C and 5% CO_2_ humidified atmosphere. AFG_1_ was diluted in DMSO (10 μg AFG_1_/μL DMSO) and added to the cultures to obtain final concentrations of 0.5 mg/L according to our previous study^29^. The concentrations of IL-6 and TNF-α were 20 ng/mL. DMSO (0.1%) was added to the A549 cells as the solvent control.

A549 cells were treated with AFG_1_ (0.5 mg/L or 2, 4, 10 mg/L) alone, IL-6 (20 ng/mL), TNF-α (20 ng/mL) or 0.5 mg/L AFG_1_ + IL-6 + TNF-α together for 3 h, 48 h, or 72 h according to the aim of the experiment.

The human monocytic leukemia cell line THP-1 was obtained from resource center of Peking Union Medical College Hospital, China. Human monocytes (THP-1) derived macrophages (MΦ-THP-1) were generated by PMA treatment for 48 hours. MΦ-THP-1 cells were treated with AFG_1_ (4 mg/L) or TNF-α (20 ng/mL) for 24 h, then the cells were collected.

### Western blot

The total protein samples isolated from the lung-adenocarcinoma or control lung tissues were collected from our previous study to detect TNF-α, IL-6, E-cadherin, vimentin, Twist, MMP-2 and MMP-9 expression by Western blot^4^.

In the *in vitro* experiment, A549 or MΦ-THP-1 cells were harvested and homogenized in 100 μl lysis buffer. Total protein was extracted by centrifuging at 12,000 rpm for 30 min at 4 °C. Protein concentrations were determined using a standard bicinchoninic acid assay (BCA) kit (Thermo). Then, 30–100 μg proteins were separated by 10% SDS polyacrylamide gel electrophoresis, and proteins were transferred to polyvinylidenefluoride (PVDF) nylon membranes. After blocking with 5% non-fat milk in Tris-buffered saline and Tween-20, the membranes were incubated with TNF-α, IL-6, E-cadherin, vimentin, Twist, MMP-2, MMP-9, STAT3, p-STAT3, IKB-α, IKB-β, NF-κB p65, and SOD-2 at 4 °C overnight (dilution 1:1000). Antibody for β-actin (Santa Cruz Biotechnology, Santa Cruz, CA) was used as the control. To detect nuclear translocation of NF-κB p65, we extracted nuclear proteins using a Thermo Scientific Pierce NE-PER kit (Thermo, Waltham, MA, USA). The membranes were incubated with *p*-NF-κB p65 and histone (used as the control) at 4 °C overnight (dilution 1: 1000). The washed membranes were incubated with rabbit anti-goat IgG secondary antibody (dilution 1:2000) or mouse anti-goat IgG secondary antibody (dilution 1:5000) for 1.5 h at 37 °C. The protein signals were developed using a chemiluminesence (ECL) detection system. Images obtained were quantified using the densitometric analysis software (BIO-1D).

### RNA isolation and qRT/PCR

After treatment, A549 or MΦ-THP-1 cells were homogenized with TRIzol reagent and total RNA of A549 cells was isolated using an RNeasy-Kit (Qiagen, Hilden, Germany) according to the manufacturer’s instructions. Total RNA of lung tissues from mice model was isolated and collected in our previous study^4^.

The total RNA from mice was reverse transcribed to cDNA in a total volume of 25 μl of reaction mixture containing: 2 μg of total RNA, 0.5 μl of Oligo DT (0.5 μg/ml), 0.5 μl of RNase inhibitor (30U/μl), 2.5 μl of dNTPs (10 mM), 5 × RT buffer, and 0.4 μl of AMV (5U/μl) (Promega, Beijing, China). Transcript levels of TGF-β, IL-1, IL-6, TNF-α and CCL-2 were evaluated by real-time PCR using a SYBR@Green Kit (Invitrogen). Primers used for the amplification are described in the Table 1. The total RNA from A549 cells or MΦ-THP-1 was reverse transcribed to cDNA. Transcript levels of IL-1, IL-6, TNF-α, SOD-2, STAT3, and NF-κB as vimentin, E-cadherin, MMP-2 and MMP-9 were evaluated by real-time PCR. Primers used for the amplification are described in the Table 2. Gene expression was normalized against the housekeeping gene β-actin. Relative gene expression was calculated by the comparative ΔΔCt method according to the manufacturer’s instructions.

**Table 1:** Mouse primer sets used for qRT/PCR.

	Forward	Reverse
TGF-β	AGGGCTACCATGCCAACTTC	CCACGTAGTAGACGATGGGC
IL-1β	CGTGGACCTTCCAGGATGAG	CATCTCGGAGCCTGTAGTGC
IL-6	CGGCCTTCCCTACTTCACAA	TCTGCAAGTGCATCATCGTT
TNF-α	TGTCCCTTTCACTCACTGGC	CATCTTTTGGGGGAGTGCCT
CCL-2	GTGCTGACCCCAAGAAGGAA	TGCTTGAGGTGGTTGTGGAA
β-actin	GTACCACCATGTACCCAGGC	GCAGCTCAGTAACAGTCCGC

**Table 2:** Human primer sets used for qRT/PCR.

	Forward	Reverse
STAT3	AACGACCTGCAGCAATACCA	TCCTCACATGGGGGAGGTAG
NF-κB	CTGAACCAGGGCATACCTGT	GAGAAGTCCATGTCCGCAAT
SOD-2	CACTGCAAGGAACAACAGGC	ACCAGGCTTGATGCACATCTT
IL-1β	TCCCCAGCCCTTTTGTTGAG	GGAGCGAATGACAGAGGGTT
IL-6	TGCAATAACCACCCCTGACC	GTGCCCATGCTACATTTGCC
TNF-α	CCCAGGGACCTCTCTCTAATC	ATGGGCTACAGGCTTGTCACT
β-actin	AGCGAGCATCCCCCAAAGTT	GGGCACGAAGGCTCATCATT
E-cadherin	CTTTGACGCCGAGAGCTACA	TTTGAATCGGGTGTCGAGGG
Vimentin	AAACTTAGGGGCGCTCTTGT	GAGGGCTCCTAGCGGTTTAG
MMP-2	CCAGCTGGCCTAGTGATGAT	CTGGGTCCAGATCAGGTGTG
MMP-9	TCTATGGTCCTCGCCCTGAA	CATCGTCCACCGGACTCAAA

### Wound-healing assay

To determine cell migration, A549 cells were seeded into 6-well plates. Wounded cell monolayer was scratched with a 200-microliter pipette tip and rinsed with phosphate-buffered saline (PBS). Then, A549 cells were incubated with IL-6, TNF-α, or IL-6 + TNF-α to generate confluent cultures. The migration of the cells at the edge of the scratch was monitored at 48 h. The cells were stained and photographed. At least three independent experiments were performed.

### Transwell migration assay

A549 cells were plated in medium without serum in the top chamber of a transwell (Corning, NY). The bottom chamber contained standard medium with 10% FBS plus IL-6, TNF-α, or IL-6 + TNF-α. After 48 h of incubation, the cells that had migrated to the lower surface of the membrane were fixed with formalin, stained with crystal violet, and photographed under microscope. Cell numbers in bottom chamber were counted under a light microscope at 400×. Experiments were carried out at least three times.

### Transient siRNA knockdown

Cells (1 × 10^5^ cells/well) were seeded into 6-well plates. Transfections were carried out using Lipofectamine 2000 reagent (Invitrogen) according to the manufacturer’s protocol. The cells were cultured for 24 h to allow the knockdown. After, the cells were treated with IL-6 + TNF-α or TNF-α alone for 24 h, 48 h, or 72 h according to the design of experiments. The control siRNA, SOD-2 siRNAs, STAT3 siRNAs, and NF-κB siRNAs were obtained from Genephma, China. The siRNA sequences were shown in Table 3.

**Table 3:** siRNA sequences.

	Forward	Reverse
Control siRNA	UUCUCCGAACGUGUCACGUTT	ACGUGACACGUUCGGAGAATT
STAT3 siRNA	GCGACCUGGUGUGAAUUAUTT	AUAAUUCACACCAGGUCCCTT
NF-κB siRNA	CGCCAUCUAUGACAGUAAATT	UUUACUGUCAUAGAUGGCGTT
SOD-2 siRNA	GGGUUGGCUUGGUUUCAAUTT	AUUGAAACCAAGCCAACCCTT

### Detection of E-cadherin and vimentin by immunofluorescence assay

A549 cells were seeded in a cover-slide and transfected with control siRNA or SOD-2 siRNAs for 24 h. After 72 h of TNF-α treatment, the samples were fixed and incubated with the primary antibody of E-cadherin and vimentin at 4 °C overnight, followed by fluorescently conjugated secondary antibodies (Jackson, 1:1000). The sections of the control were treated with isotype controls by replacing the primary antibody. Images were obtained with a Zeiss LSM 710 inverted confocal microscope.

### Statistics

Results were analyzed with two-tailed ANOVA using the PRISM software, version 3.0. *In vitro* experiments were repeated at least three times. Data was analyzed by using the non-parametric Mann-Whitney U test. Significance was determined when p < 0.05, unless otherwise stated.
